# Day of surgery cancellation rate after preoperative telephone nurse screening or comprehensive optimization visit

**DOI:** 10.1186/s13741-015-0022-z

**Published:** 2015-12-10

**Authors:** Ronald P. Olson, Ishwori B. Dhakal

**Affiliations:** Department of Anesthesiology, Duke University Medical Center, Box 3094, 40 Duke Medicine Circle Dr, Durham, NC 27710 USA

**Keywords:** Preoperative assessment, Nurse screening, Day of surgery cancellation

## Abstract

**Background:**

Structured preoperative assessment has been reported to improve operating room efficiency as measured by metrics such as day of surgery cancellations (DOSCs). However, not all patients require comprehensive assessment; routine full assessments can result in unnecessary duplication of tests and investigations. Selective nurse screening under the supervision of anesthesiology may provide adequate information gathering in lower risk patients. This study is undertaken to assess if DOSC rates vary with different assessment processes.

**Methods:**

At a single academic tertiary care hospital, from Jan 2 to May 31, 2013, the consecutive patients undergoing comprehensive preoperative assessment (CPA) and nurse screening (NS), as well as the patients not assessed by the anesthesiology-supervised preoperative process, were followed for the occurrence and reason for DOSC. The operating room schedule of all elective surgery patients was analyzed to allow calculation of rates of DOSCs. Reasons for cancellations were documented as one of ten structured reasons by preoperative holding area clerical staff.

**Results:**

Overall, there were 14,893 elective surgery patients in this time period, with 183 DOSCs, giving a rate of 1.23 % (95 % CI 1.06, 1.42). Patients who received CPA numbered 5980; 29 of them had a DOSC, giving a rate of 0.48 % (95 % CI 0.33–0.70) (*P* < 0.0001 vs. no assessment). Patients receiving NS numbered 1840; 11 of them had a DOSC, giving a rate of 0.60 % (95 % CI 0.30–1.10) (*P* < 0.0001 vs. no assessment). The most common reason for cancellation was new medical condition.

**Conclusions:**

A very low DOSC rate can be achieved with a comprehensive preoperative process where some patients are selectively telephone screened by nurses, with complete assessment deferred to the anesthesiologist on the day of surgery.

## Background

Day of surgery cancellations (DOSCs) are a source of inefficiency and frustration to patients and hospital staff alike. They may be a measure of operating room efficiency (Fixler and Wright 2013; Macario 2006). While some last-minute cancellations will always occur because of changes in medical conditions, many of the factors that result in DOSC can be detected in time to avoid gaps in the surgical schedule and inconvenience to patients. Reducing DOSCs requires knowing what the causes are and whether the processes intended to reduce DOSC actually do reduce them.

One method proposed to minimize DOSC is a robust preoperative assessment process (Ferschl et al. 2005; Knox et al. 2009; Emanuel and Macpherseon 2013; Fischer 1996; Pollard et al. 1996; Bader et al. 2009; Xue et al. 2013). While traditionally this has been done directly by an anesthesiologist and/or consultants (Newman et al. 2013), in many cases it can be done at less cost by a nurse practitioner, physician assistant, or a registered nurse (Wittkugel and Varughese 2015), who is supervised by a department of anesthesiology (Kinley et al. 2002; van Klei et al. 2006). This observational study was undertaken to accurately determine the rate and reasons of DOSCs at a single academic tertiary care institution where the preoperative assessments are done primarily by non-physicians, as well as to observe the impact on DOSCs of the two complementary methods of preoperative assessment—a comprehensive clinical preoperative assessment by a nurse practitioner or physician assistant and a selective advanced nurse screening (NS) process, usually done by telephone, to see if there was a difference.

## Methods

The study was approved by the Duke University Medical Center Institutional Review Board. It was determined that because information gathered did not include personal identifiers, individual consents were not required. At an academic tertiary care hospital, for the period Jan 2 to May 31, 2013, the operating room schedules, as published in the afternoon of the day before surgery, were collated to form the denominator of total elective cases. Emergency cases are not added to these schedules. Friday afternoon was used as the cutoff for Monday surgeries. Ambulatory surgery center cases, eye center cases, and electroconvulsive therapy cases were not included as documentation of reasons for cancellation was done by different staff, and thresholds for cancellation are different. Gastroenterology suite procedures done under anesthesiology care were similarly different but were included as there was special interest in the putative benefit of an extensive preoperative assessment in this generally low-risk situation.

The study period was begun when a consistent process for recording the date, time, and reason for DOSCs had been established. Previous observations suggested that preoperative area clerks were in the best position to record an unbiased reason for cancellation, although it was not done consistently. Therefore, they had been encouraged, empowered, and monitored over the previous 6 months by their supervisors and the study personnel, to be diligent and consistent in determining and documenting the reason for cancellation. The study period ended when implementation of a new electronic health record changed data-gathering methods and resource allocation.

The date and time of all cancellations were recorded in the electronic scheduling program. These cancellations were compared with the list of completed cases, to verify that no cancellation matched to a case that was ultimately started later that day. Those confirmed DOSCs were taken as the numerator for the rate calculation.

The reasons for DOSCs were noted as one of ten standardized categories. Where the reason was not noted, or if it was noted as unknown, the electronic medical record was examined retrospectively to determine a reason if possible.

At this institution, most patients scheduled for elective surgery are assessed by either telephone NS or comprehensive preoperative assessment. The surgical clinics utilize a published criteria list to determine which patients are lower risk and therefore eligible for the NS process (see Table 1). Some patients do bypass these two processes and are assessed by the perioperative team on the day of surgery. This occurs if they are very low risk or have had adequate outside assessment and documentation or late scheduling precluded earlier assessment.

**Table 1:** Criteria for phone screening

Must be		
	-BMI < 40 and weight < 350 lbs (160 kg)	
	-Vital signs recorded within the last 60 days	
	-English speaking	
	-Age 14–65	
Acceptable co-morbidities include		
	-Allergies	< 5 food, drug, or other allergies Controlled seasonal allergies acceptable
	-Anemia	Only if from menorrhagia associated with planned surgery and Hct > 26 % (Hgb > 8 g/dl) documented <30 days
	-Seizures	Seizure-free >1 year
	-Depression	Stable on ≤ 2 meds
	-Endocrine	Stable hypothyroidism with no recent med changes
	-Diabetes	Controlled ≤ 1 oral med. Hgb A1C < 7.5 within the last 3 months
	-Hypertension	Controlled ≤ 3 meds (<170/<95)
	-Mitral valve prolapse	If asymptomatic
	-Neoplasms without organ metastasis	Head and neck, thyroid, soft tissue, orthopedic, breast, renal cell, melanoma
	-Smoker	No new productive cough, no severe COPD
Exclusions		
	-Severe systemic disease	
	-Coronary artery disease	
	-Scheduled for high-risk surgery	
	-Refuses blood transfusion (unless seen by a center for blood conservation)	

To be eligible for nurse screening, a patient must be all of the first group of criterion, may have any of the second group, but must not have any of the third group

### Comprehensive preoperative assessment (CPA)

The comprehensive preoperative assessment (CPA) involves teaching by a nurse (described below), as well as assessment and management done by either a nurse practitioner or physician assistant. It includes a complete medical history, medication reconciliation, focused examination, securing outside records, and determining and arranging indicated tests and consultations. Limited medical management such as preemptive control of pain and nausea; smoking cessation; ordering and assessing echocardiography reports; and basic optimization of diabetes, asthma, simple infections, and hypertension is performed. Follow-up is instigated as needed. Preoperative medication instructions are given. Documentation is formatted in the institutional electronic record with anesthesiology in mind as the primary user, but the information can also provide the basis for the admission history and physical evaluation by surgeons and house staff.

Basic education on anesthesia options are given, and written anesthesia consent, separate from the surgical consent, is obtained.

CPAs are done in four locations and are supervised by one physician—either an anesthesiologist or a specifically trained family physician.

### Nurse screening (NS)

A NS process entails reviewing health history, current health status, medication reconciliation, description of the logistics and expectations of the perioperative experience, and instruction on preoperative fluids. When indicated, specific instructions on bowel preparations and enhanced recovery after surgery protocols (Miller et al. 2014) are addressed. Instructions on preoperative medications are given, based on a protocol and in consultation with the attending physician. Documentation is in the same institutional electronic record as the CPA and can be used by other health care staff as appropriate.

Patients assessed by NS are intended to be a different, lower risk group of patients than those seen with CPA.

To determine the rate of DOSC for patients undergoing one of these encounters, the respective denominators were defined either by a compilation of the daily schedules of the preoperative assessment clinic or by the records of NS contacts. The numerators for rates were determined by scanning the list of all CPAs and NS encounters, with the dates, and matching forward to any DOSC occurring for that patient in the 30 days after the preoperative assessment. This is thus a cohort study of three groups: CPAs, NS, and other preoperative assessments.

### Statistical analysis

DOSCs by cancellation reason and surgical service were presented as group frequencies and percentages. DOSC rate was calculated by dividing the number confirmed DOSC cases (numerator) by the total number of scheduled cases (denominator) in the corresponding category. Descriptive comparisons of rates between groups were made by using chi-square or Fisher’s exact test, as appropriate. The proportions and 95 % confidence intervals (CI) were computed by employing the Clopper-Pearson exact method. Statistical significance was set at *P* < 0.05 (two-sided). The analyses were performed with SAS statistical software (version 9.4, SAS institute Inc, Cary, NC).

## Results

### Rates of cancellations

From Jan 2 to May 31, 2013, the number of patients who were on the surgical schedule for the main operating rooms, offsite locations where anesthesiology care occurs, and the gastroenterology suite, as of the afternoon posting on the day before surgery, totaled 14,893. There were 183 DOSCs, giving an overall rate of 1.23 % (95 % CI 1.06, 1.42). The rate for medical reasons (inadequate investigation or optimization and new or changed medical condition) was 0.63 %.

In this time period, 62 % of the patients having elective surgery were assessed with one of the two preoperative processes—NS or CPA. The others were assessed by the perioperative team on the day of surgery.

Ninety-eight percent of elective cases were admitted on the day of surgery.

Patients who had no assessment by one of the structured preoperative processes numbered 7073; 143 of them had a DOSC, giving a rate of 2.02 % (95 % CI 1.71–2.38).

Patients who received CPA numbered 5980; 29 of them had a DOSC, giving a rate of 0.48 % (95 % CI 0.33–0.70) (*P* < 0.0001 vs. no assessment). Medical reasons (inadequate investigation or optimization and new or changed medical condition) accounted for 13 of these, giving a rate of 0.22 %.

Patients receiving NS numbered 1840; 11 of them had a DOSC, giving a rate of 0.60 % (95 % CI 0.30–1.10) (*P* < 0.0001 vs. no assessment). Eight of these were due to medical reasons giving a rate of 0.43 % (see Table 2). Of the preoperative assessments in this period, 23.5 % (12.5 % of the total scheduled cases) were done as a NS process. Application for NS was denied in 187 cases because the criteria were not met.

**Table 2:** Reasons for day of surgery cancellations

Reasons for cancellation	Overall		Comprehensive preoperative assessment		Nurse screening	
	Number	%	Number	%	Number	%
Inadequate investigation or optimization	14	0.1	3	0.05	1	0.03
New or changed medical condition	91	0.6	14	0.24	2	0.06
NPO guidelines not met	7	0.05	2	0.03	1	0.03
OR or equipment unavailable, schedule changes	25	0.2	3	0.05	2	0.06
Patient-initiated cancellation	20	0.1	1	0.02	3	0.09
Pt transportation/logistics breakdown	7	0.05	1	0.02	2	0.06
Surgeon unavailable	3	0.02	0	0.00	0	0.00
Surgery no longer needed	27	0.2	2	0.03	0	0.00
Financial issues	2	0.01	1	0.02	0	0.00
Unknown/not stated	17	0.1	4	0.07	2	0.06
Grand total	212	1.4	31	0.5	13	0. 4

The overall DOSC rate for gastrointestinal cases was 0.69 %. Of the 385 cases seen by CPA or NS, there was only one DOSC (0.3 %).

Of the CPAs, 1982 were done on the day of or the day before surgery; 4 of these became DOSCs giving a rate of 0.20 % (95 % CI 1.71–2.38). Of the NS assessments, 385 were done on the day of or the day before surgery; 1 of these became DOSC giving a rate of 0.26 % (95 % CI 0.01–1.43). The absolute risk difference in DOSCs on the day of or the day before surgery between comprehensive assessments and NS was not statistically significant—0.06 % (95 % CI −0.60–0.49), *P* = 0.59.

Comparison of pediatric vs. adult patients showed no difference in the DOSC rates or the reasons. DOSC rates by surgical service are shown in Table 3. American Society of Anesthesiologists (ASA) Physical Classification Spectrum is shown in Table 4.

**Table 3:** Day of surgery cancellations by service

Service	DOSC	Cases	Rate (%)
Cardiac	9	2389	0.38
Dentistry	4	134	2.99
Dermatology	0	127	0.00
Gastrointestinal	6	1112	0.54
General	70	2372	2.95
Gynecology	4	558	0.72
Medicine	0	469	0.00
Neurosurgery	13	1475	0.88
Otolaryngology	11	839	1.31
Orthopedic	50	2175	2.30
Pediatric	16	498	3.21
Hematology	1	176	0.57
Plastic	12	556	2.16
Radiology	0	198	0.00
Thoracic	5	851	0.59
Urology	11	1101	1.00
Others < 50 cases per service	0	103	0.00
Grand total	212	15,133	1.40

**Table 4:** American Society of Anesthesiologists (ASA) Physical Classification Spectrum

ASA classification	All surgery^a^ (%)	CPA (%)	NS (%)
1	5.9	4.0	29.1
2	34.3	37.6	60.2
3	45.6	51.0	8.6
4	7.0	4.4	0.1
1–4E	7.3	3.0	2.1

^a^The dataset used to calculate “All surgery” was different than the denominator dataset in this study

## Discussion

DOSC causes are multifactorial, as are solutions, but structured preoperative screening and assessment programs have been shown to reduce DOSCs (Ferschl et al. 2005; Knox et al. 2009; Emanuel and Macpherseon 2013; Pollard et al. 1996), as well as reduce operating room startup times, preoperative testing, postoperative complications, and workload for surgical clinic staff (van Klei et al. 2006). In a sometimes fragmented health care system, the preoperative assessment may be one of the few opportunities to identify long-term health issues (Kain et al. 2015) and initiate management. But while there is good evidence to support such programs, they can be expensive (Qiu et al. 2006), and reimbursement for this process may increasingly become part of a global admission fee. Therefore, it is useful to analyze how these preoperative processes can be modified to reduce costs, duplication, and inconvenience but still to reliably triage unoptimized patients to more intensive investigation, to provide consistent assessment and documentation, and to minimize DOSCs. An increasingly common process is one where preoperative information gathering and education, in selected lower risk cases, are done by a nurse, often by telephone (Wittkugel and Varughese 2015; Kinley et al. 2002; Boudreau and Gibson 2011; Dexter et al. 2014). A previous study of these processes at this institution showed equally high patient satisfaction with NS and CPA (Olson and Bock 2011). Because of the success of this process, the thresholds are sometimes expanded to more complicated patients; as this happens, it is important to analyze whether downstream effects, such as DOSCs, are affected negatively.

Determining a rate of DOSCs is challenging (Dexter et al. 2005; Ehrenfeld et al. 2013) because cancellations and cases are increasingly electronic, changing, and difficult to capture after the fact. The iteration used in this study is similar to previous studies (Argo et al. 2009; Leslie et al. 2012; van Klei et al. 2002), being the schedule published in the afternoon of the day before surgery.

This study shows that the overall DOSC rate at this institution is only 1.23 %. That the preoperative process is driving at least part of the low rate is suggested by the fact that the CPA rate of 0.48 % and the NS rate of 0.60 % are both lower than the overall rate of 1.23 %.

### Comparison with other studies

Comparison of DOSC rates between institutions and points in time must be done cautiously (Dexter et al. 2005) and in general terms only. But comparing the rate in this study to other studies does warrant some comment, as the rate in this study is one of the lowest published, with the methodology and numbers, though modest, are more substantial than most.

It has been suggested that a reasonable target for DOSC is a rate of less than 5 % (Macario 2006). Academic medical centers have been reported to have DOSC rates twice as high as private or smaller hospitals (Schuster et al. 2011). In all published cases of DOSC rates < 5 %, a significant part of the success has been attributed to a structured preoperative process (see Table 5).

**Table 5:** Comparable published studies of DOSC rates

Study	Year	Subjects	DOSC rate (%)	Most common		Preop assess	Notes
				Reason	Absolute rate (%)		
Fischer (Fischer 1996)	1996	7485	0.2	Medical reasons	0.2	Yes	Cancellations after patient in operating suite
van Klei (van Klei et al. 2002)	2002	8466	4.6	Logistical reasons	2.7	Yes	Patients admitted preoperatively, mean 1.5 days
			0.9	Medical reasons	0.9	Yes	4.6 % is overall DOSC rate, 0.9 % for medical reasons
Trentman (Trentman et al. 2010)	2010	12,176	2.0	New condition	0.7	Yes	Expandable block time scheduled
Hussein (Hussain and Khan 2005)	2005	8526	4.0	Not stated	4.2	Yes	Pakistan
Hovlid (Hovlid et al. 2012)	2012	3021	4.9	Schedule overrun	Not stated	Yes	Norwegian community hospital
Gillen (Gillen et al. 2009)	2009	27,632	5.0	New/unknown medical condition	Not stated	Not stated	
Xue (Xue et al. 2013)	2013	2751	7.5	Inadequate preop preparation	2.2	No	
Pollard (Pollard et al. 1996)	1996	561	6.6	Medical reasons	2.2	Yes	Outpatient surgery
Leslie (Leslie et al. 2012)	2012	19,141	8.1	Process	4.7	Not stated	Canada, urological procedures
Argo (Argo et al. 2009)	2009	329,784	12.4	Patient related	4.3	Yes	Administrative data VA hospitals
Pollard (Pollard and Olson 1999)	1999	529	13.2	Insufficient OR time	2.8	Yes	Prospective

Studies at Ambulatory Surgery Centers are not included. The most common reason is expressed as a percent of total cases

*Preop assess* Institutional Preoperative Assessment Process, *VA* Veterans Administration

Fischer described the lowest published rate (Fischer 1996). That study differed from others in that it enumerated only cancellations occurring just before the patient entered the operating room. The rate for medical reasons was 0.2 %, which is similar to the 0.22 % rate for similar reasons in this study.

van Klei reported a rate of 0.9 % for medical reasons. This study from the Netherlands in the late 1990s was at a hospital where, even after the introduction of the outpatient preoperative evaluation clinic, the average preoperative hospital stay was 1.5 days (van Klei et al. 2002). In our study, the DOSC for the comparable categories was lower—being 0.63 % overall, 0.22 % if assessed with CPA, and 0.43 % for selective NS.

Prospective studies tend to have higher DOSC rates than retrospective ones (Pollard and Olson 1999). In this study, while the list of scheduled cases, CPA, and NS was collated retrospectively, it was used in a prospective fashion to determine which of the cases assessed preoperatively became a DOSC.

### Reasons for cancellation

Because causes of DOSC are often multifactorial (Leslie et al. 2012), statistical analysis of the causes is fraught with difficulty (Leslie et al. 2012). Fixing one cause may not result in an immediate improvement; nevertheless, it will be usually useful for each institution to determine the most common reasons for DOSC and tackle those first.

As a balance of ease of recording and amount of detail, we chose 10 categories similar to previous studies (Xue et al. 2013; Gillen et al. 2009; Trentman et al. 2010). Although the reason for cancellation may be described differently by different staff members, we previously observed that the preoperative area clerks were the most consistent and probably least biased source of cancellation documentation. The fact that “unknown” continued to be a common reason shows that it is still an imperfect source. There is currently no validated instrument for description of cancellations. Inter-observer or intra-observer reliability was not assessed.

The most common reason for cancellation was a new medical condition. This is consistent with many other studies (Emanuel and Macpherseon 2013; Pollard et al. 1996; Trentman et al. 2010; Garg et al. 2009).

In this study, there was no clinically significant difference in the DOSC rate between services. Other studies have reported higher relative rates for general surgery (Argo et al. 2009).

Anecdotally, doing a preoperative assessment the day before surgery is considered suboptimal as the opportunity to optimize is limited. However, this study does not show an increased rate of DOSCs for patients assessed the day before or the day of surgery. At this institution, there are staff and resources available to coordinate last-minute assessments and investigations if needed. At some institutions, these resources may not be available, and cancellation would be necessary. A recent study of cancellations of inpatients also suggested that late assessments were not a major contributor to DOSCs (Dexter et al. 2014).

Patients undergoing gastroenterology endoscopy procedures had a low rate of DOSC. One of these DOSCs had been assessed by NS on the scheduled day of surgery and cancelled because surgery was no longer needed. So while this study suggests that there is room for improvement, possibly with preoperative assessments, the rate is already so low that justification of preoperative assessments in this group will need to include reasons other than prevention of DOSC—such as preventing delay of surgical start time. Further study on the effect of preoperative assessments on delay of surgery start time is needed.

### ASA classification

The threshold for NS was fairly inclusive—60 % were ASA 2 and 9 % were ASA 3 classification. The mix of ASA classification of patients assessed with CPA was of slightly higher complexity than other studies which describe the classification (Ferschl et al. 2005) (see Table 4).

### Shortcomings

This is a cohort study with subgroups; therefore, comparison of rates can only be done in a very general, observational fashion. The demographics and preoperative morbidity of the CPA, NS, and non-assessed groups were not compared.

The denominators of this study are snapshots of a constantly changing number. The posted schedule the afternoon before surgery will differ from the completed case list, not only by the DOSCs and by add-ons but also by cases moved to other sites. Cases that were known to be cancelled by the surgeon and the patient more than 24 h before scheduled time, but not known to the scheduling staff, will have been included as DOSCs.

It is not possible to determine how many DOSCs, potentially a result of inadequate preoperative process, were averted by cooperative schedule adjustments or urgent arrangement of medical investigations that satisfied concerns and allowed a postponed start. The study did not capture who made the decision to cancel the case.

The study included primarily outpatients. In the time period of the study, 98 % of elective cases where admitted the day of surgery. Emergency cases from either the emergency department or the wards were not included. Thus, it cannot be compared to studies that concentrate on inpatients (Epstein and Dexter 2015).

Some authors have described that cancellation rates should be compared in batches, because one cancellation may result in others (Dexter et al. 2005). In this study however, there was rarely more than one cancellation per day, so such interactions were not likely a common issue.

### Cost of DOSC

Calculating the financial impact of cancellations is challenging, as cancellations actually decrease some variable costs, and the potential lost income varies by the contribution margin of the procedure (Macario et al. 2001).

The value of a low DOSC also includes patient satisfaction, less delays in the preoperative holding area, and less staff frustration (Bader et al. 2009). It is difficult to assign a monetary value to these issues.

A low DOSC rate is perhaps more a marker of medical optimization and efficiency than a determinant of those factors. As long as the rate of cancellations is relatively predictable, it is possible to plan for it and thus not impact operating room efficiency. Therefore, a more practical goal is to maintain a low DOSC rate commensurate with the efficiency of other aspects of a particular institution’s operating room management but with the least resources and patient inconvenience required to produce safe and efficient perioperative care.

### Outside referral vs. surgical home model of care

Many facilities operate without a comprehensive preoperative process by deferring the preoperative process to clinicians outside the institution. This will minimize the inconvenience and cost of such preparation from the institution’s perspective but not necessarily reduce inconvenience and cost from the perspective of patients or insurers.

However, it is likely that in other systems—such as single payer or Accountable Care Organizations—a coordinated, comprehensive perioperative care would prove to be a more cost-effective and patient-centered approach (Song et al. 2014). Studies are needed which compare overall charges in the perioperative period in a system with comprehensive coordinated perioperative care (surgical home model) vs. overall charges in a more traditional system with routine protocols for testing, and outside consultations are needed. The increase of all-payer claims databases should allow assessment of this (Peters et al. 2014). The state where this study was conducted does not yet have such a database.

There is much recent interest in anesthesiology involvement in the perioperative surgical home (Dexter and Wachtel 2014), but anesthesiologists are probably best utilized as directors of protocols and care as opposed to the actual delivery. This study shows that such information gathering and basic medical management can be done effectively and efficiently by advanced care clinicians and nurses, albeit with strong support, backup, and continual education from anesthesiology.

## Conclusions

A DOSC rate for all causes of less than 2 % is achievable at a tertiary care academic hospital where a CPA and management process are in place, supervised by the department of anesthesiology, but where a significant proportion of those assessments are done by a selective NS process.

## Abbreviations

CI: confidence interval
CPA: comprehensive preoperative assessment
DOSCs: day of surgery cancellations
NS: nurse screening
